# Characterization of Rice NADPH Oxidase Genes and Their Expression under Various Environmental Conditions

**DOI:** 10.3390/ijms14059440

**Published:** 2013-04-29

**Authors:** Gang-Feng Wang, Wen-Qiang Li, Wen-Yan Li, Guo-Li Wu, Cong-Yi Zhou, Kun-Ming Chen

**Affiliations:** 1 State Key Laboratory of Crop Stress Biology in Arid Areas, College of Life Sciences, Northwest A&F University, Yangling 712100, Shaanxi, China; E-Mails: xibeiwgf@nwsuaf.edu.cn (G.-F.W.); wqli@nwsuaf.edu.cn (W.-Q.L.); liwenyan@nwsuaf.edu.cn (W.-Y.L.); 2 College of Agriculture and Biotechnology, Zhejiang University, Hangzhou 310058, Zhejiang, China; E-Mails: glwoo@163.com (G.-L.W.); zhoucongyi_112@163.com (C.-Y.Z.)

**Keywords:** NADPH oxidase (Nox), phylogenetic analysis, gene expression, environmental stress, rice (*Oryza sativa*)

## Abstract

Plasma membrane NADPH oxidases (Noxs) are key producers of reactive oxygen species under both normal and stress conditions in plants. We demonstrate that at least eleven genes in the genome of rice (*Oryza sativa* L.) were predicted to encode Nox proteins, including nine genes (*OsNox1*–*9*) that encode typical Noxs and two that encode ancient Nox forms (ferric reduction oxidase 1 and 7, *OsFRO1* and *OsFRO7*). Phylogenetic analysis divided the Noxs from nine plant species into six subfamilies, with rice *Nox* genes distributed among subfamilies I to V. Gene expression analysis using semi-quantitative RT-PCR and real-time qRT-PCR indicated that the expression of rice *Nox* genes depends on organs and environmental conditions. Exogenous calcium strongly stimulated the expression of *OsNox3*, *OsNox5*, *OsNox7*, and *OsNox8*, but depressed the expression of *OsFRO1*. Drought stress substantially upregulated the expression of *OsNox1*–*3*, *OsNox5*, *OsNox9*, and *OsFRO1*, but downregulated *OsNox6*. High temperature upregulated *OsNox5*–*9*, but significantly downregulated *OsNox1*–*3* and *OsFRO1*. NaCl treatment increased the expression of *OsNox2*, *OsNox8*, *OsFRO1*, and *OsFRO7*, but decreased that of *OsNox1*, *OsNox3*, *OsNox5*, and *OsNox6*. These results suggest that the expression profiles of rice *Nox* genes have unique stress-response characteristics, reflecting their related but distinct functions in response to different environmental stresses.

## 1. Introduction

Plasma membrane NADPH oxidases (Noxs) are major sources of reactive oxygen species (ROS) production under both normal and stress conditions in plants [1,2]. Seven members of the NADPH oxidase family have been identified in animals: Nox1, Nox2, Nox3, Nox4, Nox5, Duox1, and Duox2 [3,4]. All Nox/Duox enzymes contain six membrane-spanning domains, two hemes, and conserved motifs involved in NADPH and FAD binding. In addition, Nox5 contains four calcium-binding EF-hand motifs in its *N* terminus, whereas Duox proteins contain an additional transmembrane domain, a peroxidase-like domain, and two EF-hand motifs [4]. Multiple homologs of Nox have been identified in plants [3], with ten genes in Arabidopsis genome [2]. However, all these plant Noxs belong to Nox5-like homolog of animals, and no ancestral-type Nox homologs or Duox homologs (p47^phox^, p67^phox^, or p22^phox^) have been found in plants [3].

The functions of Noxs are closely associated with the production and accumulation of ROS in plants exposed to environmental stress conditions [5–8]. During biotic or abiotic stresses, plants produce and accumulate more hydrogen peroxide (H_2_O_2_) to ease the stresses, which can be blocked by diphenylene iodinium (DPI), an important inhibitor of Noxs [9–11]. Hao *et al.* [12] found that Noxs can decrease nickel-induced oxidative stress in wheat seedling roots. *Arabidopsis thaliana* mutants lacking respiratory burst oxidase homologue D and F (*AtrbohD* and *AtrbohF*) Nox genes generate less H_2_O_2_ and are more susceptible to pathogens than wild-type plants [13]. These results implicate the importance of Noxs in plant stress tolerance. Moreover, plant Noxs also have other diverse functions in plant growth and development regulation. They participate in the development of plant immunity [14], polar growth of root hairs and pollen tubes [15–18], ABA-mediated stomatal closure [19,20], apoptotic cell death [21,22], and the control of cell differentiation and growth [23]. Plant Noxs are involved in several signaling pathways including MAPK [24], CDPK [25,26], RACK [27], phosphatidylinositol [28], phospholipase Dα1 and phosphatidic acid [20], Ca^2+^ [16,29], nitric oxide (NO) [30], cGMP [31], and extracellular ATP [32]) as well as salicylic acid, jasmonic acid, and ethylene [11] signal transduction. Therefore, Noxs (Rbohs) have been considered important molecular “hubs” during ROS-mediated signaling in plants [33] that play vital roles in both plant stress response and normal growth and development. However, the ROS signaling cascade and the regulatory mechanism of Noxs in ROS production during plant stress tolerance remain to be determined at the molecular and physiological levels.

Rice (*Oryza sativa*) is a worldwide staple crop, necessitating a clear understanding of its developmental characteristics and stress tolerance mechanisms. However, the functions of rice *Noxs* and their regulatory mechanisms in response to environmental stress remain largely unknown, although a homolog of the mammalian *gp91^phox^* gene has been identified [34]. At least nine *Nox* genes exist within the rice genome and some small Rac GTPases participate in the regulation of Nox activity in rice [35]. A direct interaction between OsRac1 and the *N*-terminal extension of OsNox2 (OsRbohA or OsRbohB) may be essential to Nox activity modulated by the cytosolic Ca^2+^ concentration in plants [35]. In addition, the rice OsNox2 and OsNox6 (OsRbohE) participate in ROS-dependent plant immune responses [36]. However, the molecular functions of most rice Noxs under different environmental conditions remain to be determined. Here, we report the characterization of the rice *Nox* gene family and their expression profiles in response to drought, high temperature, salt stress, and changes in environmental calcium.

## 2. Results

### 2.1. Identification and Domain Composition of Nox Proteins in Rice

**Figure 1:**
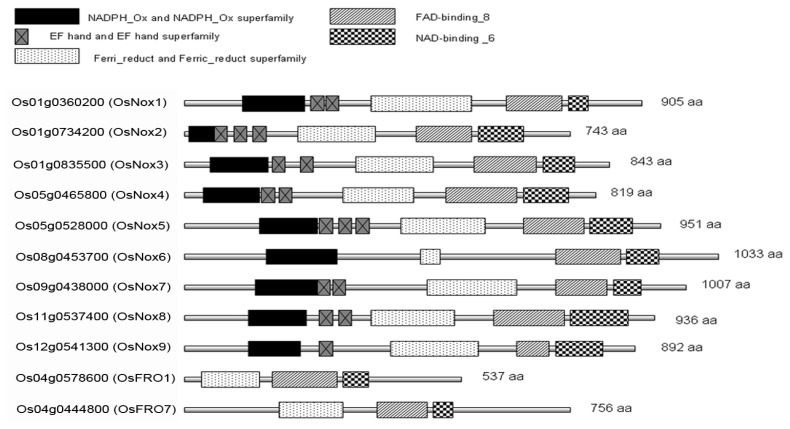
Domain compositions of rice Noxs. Nine genes encoding typical Nox proteins (*OsNox1–9*) and two encoding ancient Nox forms (*OsFRO1* and *OsFRO7*) in rice genome. Only major domains were presented here based on our database searches in NCBI (http://www.ncbi.nlm.nih.gov/), GRAMENE (http://www.gramene.org/Oryza_sativa/Info/Index), and Prosite (http://prosite.expasy.org/) databases.

In rice genome, nine genes were predicted to encode typical Nox proteins (*OsNox1*–*9*) and two genes predicted to encode ferric reduction oxidase 1 and 7 (*OsFRO1* and *OsFRO7*) were considered ancient forms of Noxs (Table 1). Among the nine typical rice Nox proteins, the smallest was OsNox2 (745 amino acids, 85.3 kDa) and the largest was OsNox6 (1033 amino acids, 115.0 kDa). The two OsFRO proteins, particularly OsFRO1 (537 amino acids), were smaller than the nine typical Noxs. Although the Nox proteins had significantly different sizes, their major functional domains were similar (Figure 1). All nine Nox proteins contained NADPH_Ox, Ferri_reduct, FAD-binding_8, and NAD-binding_6 domains, and all except OsNox6 contained one to three EF-hand Ca_2+_-binding motifs. In contrast, the two OsFROs lacked the NADPH_Ox domain and EF-hand motif. NADPH_Ox domain is the fundamental domain in respiratory burst NADPH oxidase proteins and is responsible for production of ROS as a defense mechanism in plants. This domain tends to occur to the *N*-terminus of EF-hand motifs, suggesting a direct regulatory effect of Ca_2+_ on the activity of the NADPH oxidases in plants [37]. The different numbers of EF-hand motifs among the rice Nox proteins may relate to different functions or activities in the regulation of rice development and/or environmental stress responses. Ferri_reduct domain is a ferric reductase like transmembrane component, which is required for cell surface ferric reductase activity [37]. However, NAD- and FAD-binding domains participate in membrane electron transfer, which occurs from NADPH to FAD to the heme of Cytb to oxygen leading to superoxide formation [37]. As can be seen from Figure 1, these major domains of rice Noxs distribute in different places with different sizes in the various Nox sequences.

### 2.2. Evolution and Phylogenetic Distribution of Rice Nox Proteins

**Figure 2:**
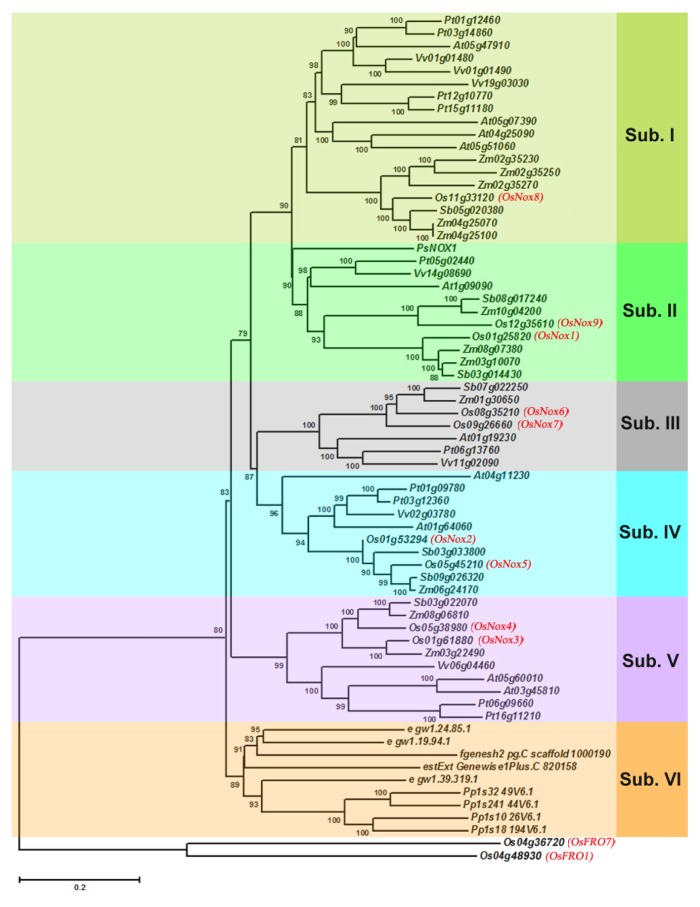
Phylogenetic relationship of Noxs in nine plants. HMM profiles of Nox proteins were used to identify Nox-encoding genes from the complete protein sets of rice and eight other plants using hmmsearch (*E* < 1 × 10^−5^) implemented in HMMER version 2.3.2 (http://hmmer.janelia.org/). The collected sequences were aligned using ClustalW v2.0 (http://www.ebi.ac.uk/Tools/webservices/services/msa/clustalw2_soap) and the unrooted phylogenetic tree was constructed using PhyML v3.0 (http://www.atgc-montpellier.fr/phyml/) with the maximum likelihood method. *OsNoxs* and *OsFROs* were indicated in red. *Sub.*, subfamily.

Hidden Markov model (HMM) profiles of Nox proteins were used to identify Nox-encoding genes from complete protein sets for rice and eight other representative plants (*Physcomitrella patens*, *Selaginella moellendorffii*, *Picea sitchensis*, *Sorghum bicolor*, *Zea mays*, *Arabidopsis thaliana*, *Populus trichocarpa*, and *Vitis vinifera*). A total of 65 proteins were recognized and aligned on a HMM phylogenetic tree (Figure 2). Two rice ferric reduction oxidases, OsFRO1 and OsFRO7, were also aligned on the phylogenetic tree as an additional group. The plant Nox proteins could be grouped into six subfamilies. Subfamilies I to V exist in monocots and dicots, while subfamily VI exists only in lower plants such as mosses and lycophytes. No algal Nox homologs were found in our database searches.

As in *Arabidopsis*, Nox proteins in rice were distributed among subfamilies I to V (Figure 2, red). OsNox8 (Os11g33120) belongs to subfamily I, thus would be the most phylogenetically recent Nox protein. OsNox9 (Os12g35610) and OsNox1 (Os01g25820) belong to subfamily II, OsNox6 (Os08g35210) and OsNox7 (Os09g26660) belong to subfamily III, and OsNox2 (Os01g53294) and OsNox5 (Os05g45210) belong to subfamily IV. OsNox4 (Os05g38980) and OsNox3 (Os01g61880) were assigned to subfamily V, and are thus predicted to be more phylogenetically ancient proteins.

### 2.3. Expression Profiles of Rice *Nox* Genes in Different Tissues

**Figure 3:**
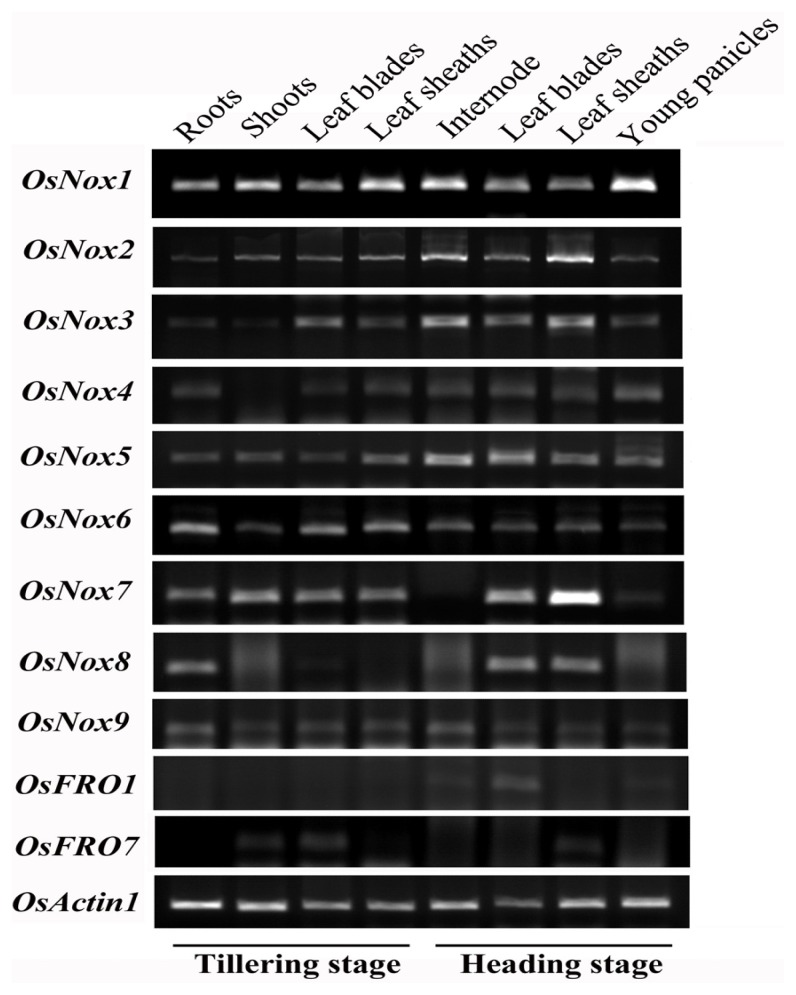
Expression profiles of rice *Nox* genes in various developmental tissues. Total RNA was extracted from various organs of rice plants grown in paddy field under normal growth conditions. Semi-quantitative RT-PCR analysis was conducted to detect the *Nox* genes expression.

To study spatio-temporal expression patterns of rice *Noxs*, total RNA was extracted from roots, shoots leaf blades and leaf sheaths at tillering stage, and uppermost internode, leaf blades, leaf sheaths and young panicles at heading stage. Semi-quantitative RT-PCR analysis revealed that *OsNox1*, −*2*, −*5*, −*6* and −*9* were ubiquitously expressed in all the tissues examined (Figure 3). However, *OsNox3*, *OsNox4*, *OsNox7*, *OsNox8*, *OsFRO1* and *OsFRO7* showed obviously tissue-specific expression (Figure 3). The *OsNox3* and *OsNox4* had extremely low expression in shoots at tillering stage. The *OsNox7* exhibited extremely high expression in leaf sheaths, but very low expression in young panicles, and no expression was detected in the uppermost internode at heading stage. The *OsNox8* showed tissue-specific expression in roots at tillering stage and in leaf blades and sheaths at heading stage. For *OsFRO1*, however, mRNA accumulations were detected only in uppermost internode, leaf sheaths and young panicles of heading stage with extremely low levels. In addition, the *OsFRO7* were expressed at low level in shoots and leaf sheaths of tillering stage and leaf sheaths of heading stage. It should be noticed that some *Nox* genes had very low expression in rice. Their expression only could be detected by semi-quantitative PCR at very high reaction cycles (Table S1), especially for *OsNox9*.

### 2.4. Expression of Rice *Nox* Genes under Reduced and Increased Calcium Conditions

**Figure 4:**
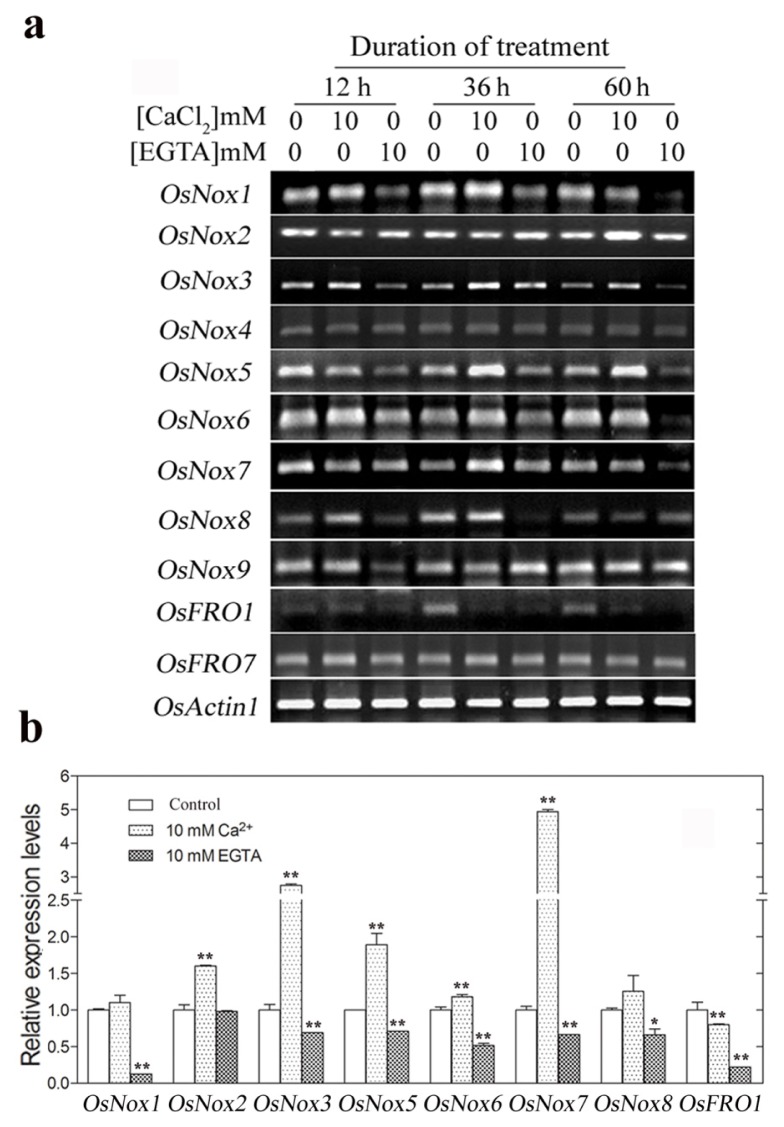
Expression levels of rice *Nox* genes under CaCl_2_ and EGTA treatment conditions. Ten-week-old plants were transferred to nutrient solution alone (control) or containing 10 mM CaCl_2_ or 10 mM EGTA for up to 60 h. Total RNA was isolated from leaves of three independently treated plants. (**a**) Semi-quantitative RT-PCR analysis of rice *Nox* genes expression at 12, 36, and 60 h with 10 mM CaCl_2_ or 10 mM EGTA treatment; (**b**) Real-time qRT-PCR analysis of rice *Nox* genes at 36 h with 10 mM CaCl_2_ or 10 mM EGTA treatment. *OsNoxs* gene expression levels were normalized to that of *OsActin1* and relative expressions were compared with that of control plants; Means values were obtained from three independent PCR amplifications. Error bars indicate SD. The significant difference in statistics between the control and treatments was carried out with one-way ANOVA analysis. *p < 0.05; **p < 0.01.

Since Ca^2+^ is well known to function as signaling molecules mediating gene expression modifications, we evaluated whether changes in environmental Ca^2+^ concentration influence the expression of *OsNox* and *OsFRO* genes. Neither addition of exogenous Ca^2+^ (10 mM) nor blocking of endogenous apoplastic Ca^2+^ with EGTA (10 mM) changed the mRNA expression levels of *OsNox4* or *OsFRO7* (Figure 4a). However, expression of *OsNox1*, *OsNox2*, *OsNox3*, *OsNox5*, *OsNox6*, *OsNox7*, and *OsNox8* were upregulated by exogenous Ca^2+^ treatment and downregulated by deprivation of endogenous apoplastic Ca^2+^ by EGTA chelation. Expression of *OsNox9* was only decreased by EGTA at 12 h. In particular, exogenous Ca^2+^ dramatically stimulated expression of *OsNox3* and *OsNox7* (2.7- and 4.9-fold, respectively) compared to controls at 36 h (Figure 4b). In contrast, both Ca^2+^ addition and deprivation caused a decrease in expression of *OsFRO1* (Figure 4a,b).

### 2.5. Expression of Rice *Nox* Genes under Drought Conditions

**Figure 5:**
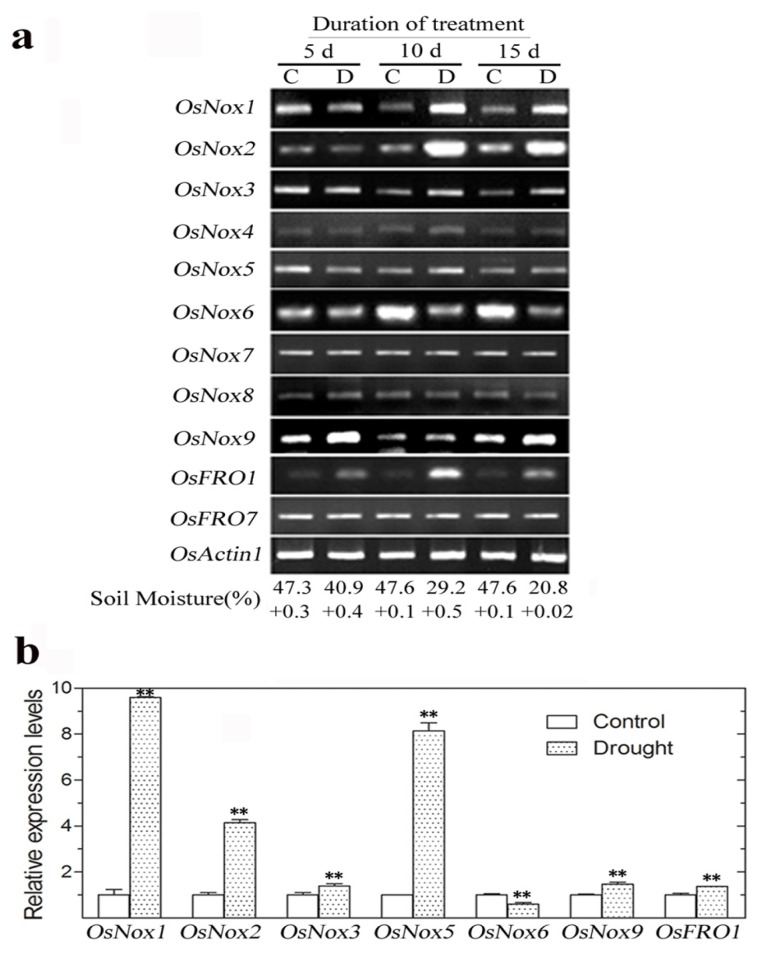
Expression levels of rice *Nox* genes under drought stress conditions. Ten-week-old plants were grown without water for up to 15 days and total RNA from leaves of three independent treatments were isolated for gene expression analysis. (**a**) Semi-quantitative RT-PCR analysis of rice *Nox* genes expression at 5 days, 10 days and 15 days drought treatment, respectively. C, control; D, drought treatment; Soil moisture (%), mean ± SD (*n* = 3); (**b**) Real-time qRT-PCR analysis of rice *Nox* genes expression at 10 d drought treatment. *OsNoxs* gene expression levels were normalized to that of *OsActin1* and relative expressions were compared with that of control plants; Means values were obtained from three independent PCR amplifications. Error bars indicate SD. The significant difference in statistics between the control and treatments was carried out with one-way ANOVA analysis. * *p* < 0.05; ** *p* < 0.01.

Differential expression profiles of *OsNox* and *OsFRO* genes under drought stress were determined after withholding water from 10-week-old plants for 5, 10 or 15 days. *OsNox1*, *OsNox2*, *OsNox3*, *OsNox9*, and *OsFRO1* expression levels were increased at 10 and 15 days drought treatment (Figure 5a), with real-time qRT-PCR analysis showing 9.6-, 4.1-, 1.4-, 1.5-, and 1.4-fold increases, respectively, compared to the control at 10 days treatment (Figure 5b). *OsNox5* expression was also significantly upregulated (8.1 fold) by drought compared to the control at 10 days (Figure 5b). In contrast, *OsNox6* expression was downregulated (1.69-fold) by drought compared to control at 10 days (Figure 5b). *OsNox4*, *OsNox7*, *OsNox8*, and *OsFRO7* showed no changes in expression under these drought stress conditions.

### 2.6. Expression of Rice *Nox* Genes at High Temperature

**Figure 6:**
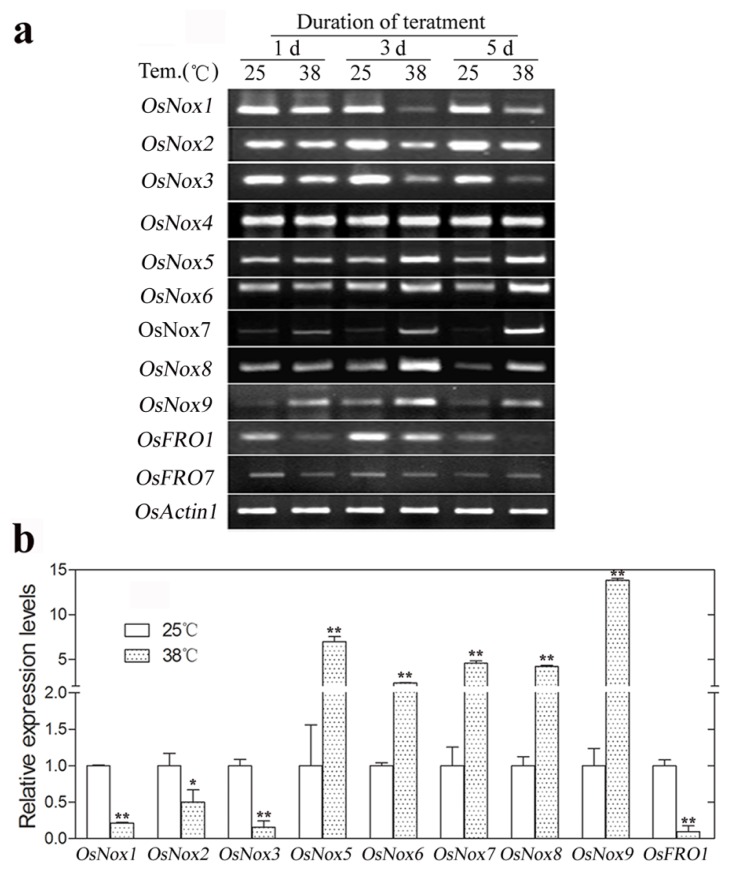
Expression of rice *Nox* genes under high-temperature conditions. Ten-week old plants were transferred to artificial chambers with 25 °C (control) or 38 °C (high-temperature) for up to 5 days. Total RNA isolated from leaves of three independent experiments were used for gene expression analysis. (**a**) Semi-quantitative RT-PCR analysis of rice *Nox* genes at 1 day, 3 days, and 5 days high-temperature treatment; (**b**) Real-time qRT-PCR analysis of rice *Nox* genes at 3 days treatment high-temperature. *OsNoxs* gene expression levels were normalized to that of *OsActin1* and relative expressions were compared with that of control plants; Means values were obtained from three independent PCR amplifications. Error bars indicate SD. The significant difference in statistics between the control and treatments was carried out with one-way ANOVA analysis. *p < 0.05; **p < 0.01.

The expression levels of *OsNox* and *OsFRO* genes under high temperature conditions are presented in Figure 6a. *OsNox1*, *OsNox2*, *OsNox3*, and *OsFRO1* were significantly downregulated at high temperature, with real-time qRT-PCR analysis showing 4.8-, 2.0-, 6.7-, and 10.0-fold decreases, respectively, compared to controls at 3 days (Figure 6b). In contrast, expression of *OsNox5*, *OsNox6*, *OsNox7*, *OsNox8*, and *OsNox9* were substantially upregulated by high temperature (Figure 6a), with 7.0-, 2.3-, 4.6-, 4.2-, and 13.8-fold increases, respectively, in relative expression levels compared to controls at 3 days (Figure 6b). *OsNox4* and *OsFRO7* expression levels did not change under high-temperature conditions (Figure 6a).

### 2.7. Expression of Rice *Nox* Genes under High NaCl Conditions

**Figure 7:**
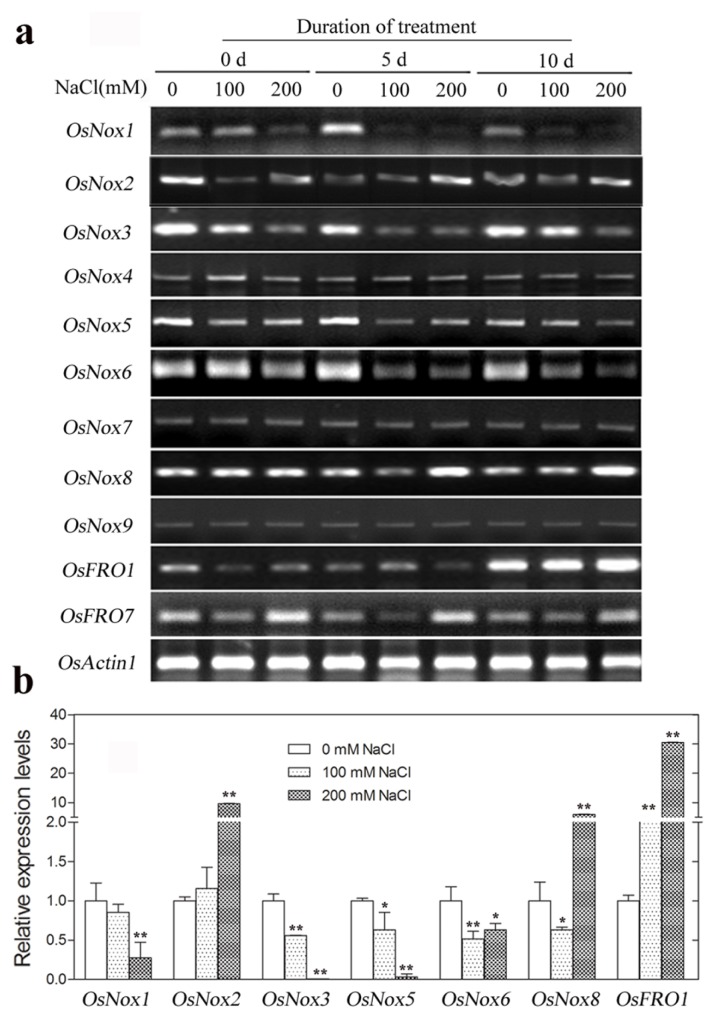
Expression of rice *Nox* genes under high salt treatment conditions. Ten-week old plants were transplanted into a solution containing 0 mM (control), 100 mM, or 200 mM NaCl for up to 10 days and total RNA isolated from leaves of three independent experiments were used for gene expression analysis. (**a**) Semi-quantitative RT-PCR analysis of rice *Nox* gene expression at 0 day, 5 days and 10 days treatment; (**b**) Real-time qRT-PCR analysis of rice *Nox* genes at 5 days treatment. *OsNoxs* gene expression levels were normalized to that of *OsActin1* and relative expressions were compared with that of control plants; Means values were obtained from three independent PCR amplifications. Error bars indicate SD. The significant difference in statistics between the control and treatments was carried out with one-way ANOVA analysis. *: *p* < 0.05; **: *p* < 0.01.

Expression of *OsNox1*, *OsNox3*, *OsNox5* and *OsNox6* were significantly downregulated by NaCl treatments (Figure 7a), with 3.7-, 100.0-, 33.3- and 1.6-fold decreases in relative expression levels, respectively, at 200 mM NaCl compared to the controls at 5 days (Figure 7b). In contrast, NaCl treatment significantly upregulated expression of *OsNox2*, *OsNox8*, and *OsFRO1* (Figure 7a), with 9.6-, 6.0- and 30.5-fold increases in relative expression levels, respectively, at 200 mM NaCl compared to the controls at 5 days (Figure 7b). *OsNox4*, *OsNox7*, *OsNox9*, and *OsFRO7* expression levels were not obviously influenced by NaCl treatment (Figure 7a).

## 3. Discussion

Many studies have shown that ROS production and Nox activity were stimulated in plants under various environmental stress conditions including drought [38], ABA and Ca^2+^ treatment [39], and nickel treatment [12]. Therefore, ROS production has been considered as an important regulatory mechanism of perception and response of plants to stresses and Noxs serve as important molecular “hubs” during ROS-mediated signalling in the plant stress responses [33]. As reviewed by Marino *et al*. [33], different Nox proteins in *Arabidopsis* serve different functions. For example, AtRbohC functions in root hair tip growth [40], AtRbohB functions in seed after-ripening [41], and AtRbohD and AtRhohF function in pathogen response and stomatal closure [20]. Although the activation mechanisms for AtRbohD and AtRbohF are similar in stress responses, AtRbohD has significantly greater ROS-producing activity than AtRbohF [42], indicating their functional diversity. In maize, four genes encoding Nox proteins have been cloned and their ABA-induced expression levels have been shown to differ [43]. Our analysis predicts that at least 11 genes in the rice genome encode Nox proteins, including nine typical Noxs and two ancient forms (Table 1, Figure 1). Of the 11 proteins, only two have been examined previously. OsNox2 (OsRbohA or OsRbohB) and OsNox6 (OsRbohE) participate in ROS-dependent plant immune responses [36] and OsNox2 is essential for cytosolic Ca^2+^-mediated Nox activity by interacting with OsRac1 protein [35].

The variety of rice Noxs and functional domain compositions implies that they have diverse functions and regulatory mechanisms in stress response and/or normal growth and development. According to the phylogenetic analysis, OsNox8 was the most recently evolved of the typical rice Noxs and was assigned to subfamily I with four *Arabidopsis* Nox proteins (Figure 2). Of these four *Arabidopsis Noxs*, *AtRbohA* (At05g07390), *AtRbohC* (At05g51060), and *AtRbohG* (At04g25090) are specifically expressed in roots, whereas *AtRbohD* (At05g47910) is expressed throughout the entire plant [2]. The deduced amino acid sequence of OsNox8 is most similar to AtRbohD, with 66% sequence identity. However, *OsNox8* mainly expressed in roots, leaf blades and sheaths of rice as reported here (Figure 3). *AtRbohD* participates in many developmental processes and stress responses, such as stomatal closure, systemic signaling, and pathogen, wound, and salt stress [2]. Expression of *AtRbohA* is sensitive to hypoxia, salt stress, and nitrogen starvation, whereas expression of *AtRbohG* is sensitive to low nitrogen and to salicylic acid treatment [2]. *AtRhohC* is involved in root hair growth [40] and signaling triggered by mechanical stimulation [16]. It currently remains unknown whether *OsNox8* has similar functions to these *AtRhohs*. The finding that *OsNox8* expression was significantly stimulated by high temperature and NaCl stress (Figures 5 and 6), implied that *OsNox8* functions in both heat and salt stresses.

OsNox1 and OsNox9 were found on the same clade of the phylogenetic tree, were assigned to subfamily II (Figure 2), and shared 59% and 58% sequence identity, respectively, with AtRbohB (At1g09090) on the same clade. *AtRhohB* is primarily expressed in germinating seeds, and knocking out this gene disrupts seed germination [41]. However, both *OsNox1* and *OsNox9* are expressed throughout the entire plant in rice (Figure 3), implying their vital role in the plant. Although the functions of *OsNox1* and *OsNox9* are not well known, this study showed that gene expression was influenced by Ca^2+^ treatment, drought, high temperature, and salt stresses, although the response patterns of the two genes were not the same (Figures 4 and 7). Both genes were strongly stimulated by drought, but *OsNox1* was downregulated and *OsNox9* was upregulated at high temperature (Figures 5 and 6). *OsNox1* expression was stimulated by calcium and reduced by EGTA, whereas *OsNox9* was unaffected by either treatment. In addition, salt stress decreased *OsNox1* expression but had no effect on *OsNox9* expression (Figure 7). These results suggest that these two genes have different but sometimes cross-talk functions in environmental stress response.

OsNox6 and OsNox7 are quite close phylogenetically, although their domain compositions are quite different (Figures 1,2). Notably, OsNox6 does not have an EF-hand motif whereas OsNox7 has two (Figure 2). The EF-hand Ca^2+^-binding motif may mediate activation of plant Noxs by directly binding Ca^2+^ [42] and participating in Rac-Rboh interactions [35,43]. Therefore, the EF-hand motif is involved in Nox-dependent ROS production because Ca^2+^ and other related signaling molecules mediate ROS production [16]. OsNox6 and OsNox7 were most similar to AtRbohE (At01g19230), with 55% and 58% amino acid sequence identity, respectively. The function of AtRbohE, however, remains to be elucidated. Although both OsNox2 and OsNox6 participate in ROS-dependent plant immune responses, OsNox2 leads to early H_2_O_2_ generation, whereas OsNox6 is responsible for late H_2_O_2_ production [36]. These results imply that activation of OsNox6 may not be directly dependent on Ca^2+^, because OsNox6 does not contain EF-hand motifs. In the present study, expression of *OsNox6* was slightly increased with exogenous Ca^2+^ and decreased with EGTA, suggesting that other Ca^2+^-related mechanisms may be involved in OsNox6 activation. Interestingly, *OsNox6* was significantly downregulated by drought and salt stresses, whereas *OsNox7* expression remained unchanged under the same conditions (Figures 5 and 7). However, *OsNox7* was significantly stimulated by Ca^2+^ treatment (Figure 4). In addition, both *OsNox6* and *OsNox7* were upregulated by heat (Figure 6), indicating their probable functional roles in heat stress response. These results suggest that *OsNox6* and *OsNox7* have different functional mechanisms for stress responses, although they are very close in evolution. The different functions between *OsNox6* and *OsNox7* might be also reflected by their different expression profiles in different tissues of the plants (Figure 3).

OsNox2 and OsNox5 were categorized in subfamily IV with distribution on the same phylogenetic tree clade (Figure 2) and they both were expressed in whole plant tissues (Figure 3). OsNox2, also called OsRbohA or OsRbohB, is involved in ROS production during the plant immune response [36], and this activity is regulated by OsRac1 and the cytosolic Ca^2+^ concentration [35]. AtRbohF (At01g64060) was most similar to OsNox2 and OsNox5 (59% and 69% sequence identity, respectively) and is a biotic stress-inducible Nox protein [13] that participates in many biological processes, such as pathogen response and stomatal closure [33]. We have recently found that knocking out *OsNox2* reduces plant growth, fertility, and drought tolerance (data not shown), indicating that *OsNox2* participates in the drought stress response as well as regulation of normal development. Very few studies have been done on *OsNox5* and its functions are unknown. In the present study, *OsNox2* expression was significantly increased by drought, salt stress, and exogenous Ca^2+^ treatment, but decreased at high temperature. *OsNox5* expression was also increased by drought and exogenous calcium treatment, but was significantly decreased by salt stress, and was increased by high temperature (Figures 5 and 7). These results suggest that these genes play important but unique roles in responding environmental stimuli such as drought, salt, and heat.

Based on their distribution on the phylogenetic tree, OsNox3 and OsNox4 appear to more ancient among the typical rice Nox proteins (Figure 2) and both protein are missing expressed in shoots of rice plants (Figure 3). They were assigned to subfamily V and shared 51%–54% sequence identity with AtRbohH (At05g60010) and AtRbohJ (At03g45810). *AtRbohH* and *AtRbohJ* are specifically expressed in stamens and pollen [2] and the latter is involved in salt tolerance [28]. The functions of *OsNox3* and *OsNox4* are unknown. In the present study, *OsNox3* was significantly upregulated by Ca^2+^ and drought, but downregulated by heat and salt (Figures 4 and 7), implying that it responds specifically to different stresses. *OsNox4* exhibited no notable changes under these environmental treatments.

Two ancient forms of rice Noxs, OsFRO1 and OsFRO7, were predicted to be transmembrane proteins of the ferric reduction oxidase family. Although OsFROs are structurally close to OsNoxs, they lack the NADPH_Ox domain found in typical OsNoxs (Figure 1). In fungi and yeast, OsFRO homologs are structurally closer to ancestral-type Noxs [3]. AtFROs are present in roots and participate in the release of insoluble iron from Fe^3+^ oxide hydrates by reducing them to the soluble transport–ready Fe^2+^ form [2]. Based on database searches and functional predictions, it is suggested that OsFRO1 is involved in iron homeostasis [44]. Indeed, Northern blot analysis indicates that *OsFRO1* is mainly expressed in leaves of Zn^−^, Mn^−^, and Cu^−^ deficient rice plants [45]. In addition, Sperotto *et al.* [46] reported that expression of *OsFRO1* in flag leaves was significantly correlated with Fe and/or Zn concentrations in seeds, suggesting a role in internal mineral transport. However, the molecular functions of *OsFROs* under different environmental conditions remain poorly understood. In the present study, *OsFRO1* could be significantly downregulated (Ca^2+^ treatment and high temperature) or upregulated (drought and salt stress), whereas *OsFRO7* was only upregulated by salt stress and was not affected by other treatments (Figures 4 and 7), indicating that *OsFRO1* and *OsFRO7* have different functions and mechanisms in stress response. Indeed, we recently found that the rice knockout mutant *osfro7* exhibits reduced tolerance to a number of environmental stresses, including drought, heat, and salinity (data not shown).

## 4. Experimental Section

### 4.1. Plant Materials and Stress Treatments

Seeds of rice cultivar Xieyou 46 (*Oryza sativa* L.) obtained from Hangzhou Seed Corporation of China were grown in a greenhouse with a day/night temperature cycle of 30 °C/25 °C and 16 h/8 h day/night conditions, with 800 μmol m^−2^·s^−1^ light intensity and 60%–65% relative humidity. For drought treatment, 10-week-old plants were grown in plastic pots without water for 5, 10 or 15 days, at which time leaves were collected for RNA isolation (see below) and soil moisture was recorded using an HH2 Moisture Meter (Qudao, Beijing, China). For calcium experiments, 10-week-old potted plants were carefully transferred to water and the soil was gently washed from the roots. The plants were then cultivated in Hoagland solution alone (control) or containing 10 mM CaCl_2_ or 10 mM EGTA for 12, 36 or 60 h. For NaCl treatment, 10-week-old plants were washed as above and grown in nutrient solution containing 0, 100, or 200 mM NaCl for 0, 5 or 10 days. For high-temperature treatment, 10-week old plants grown in plastic pots were transferred to chambers maintained at 25 °C or 38 °C for 1, 3 or 5 days. The youngest fully expanded leaves from all treatments were immediately frozen in liquid nitrogen and stored at −80 °C until further characterization. For the drought stress, NaCl treatment and high-temperature experiment, the samples were collected at 9:00 am at each sampling day. For expression analyses of rice *Nox* genes under various organs or developmental stages, rice plants were grown in paddy field under normal growth conditions.

### 4.2. Identification and Phylogenetic Analysis of Nox Family

The sequences of rice Nox and FRO proteins, including those annotated as respiratory burst oxidase proteins, were obtained from TIGR (http://rice.tigr.org/). Functional domains of these proteins were defined by the SMART database (http://smart.embl-heidelberg.de/) [47]. Protein structure and domain compositions were obtained from NCBI (http://www.ncbi.nlm.nih.gov/protein/), GRAMENE (http://www.gramene.org/Oryza_sativa/Info/Index), and Prosite (http://prosite.expasy.org/) databases. Only major domains were considered in the present study. HMM profiles (PF08414, PF08022, PF08030, and PF01794) were used to identify *Nox*-encoding genes from the complete protein set of rice (TIGR v6.1) and eight other plants, viz *Physcomitrella patens* (*Pp*), *Selaginella moellendorffii* (*Sm*), *Picea sitchensis (Ps)*, *Sorghum bicolor* (*Sb*), *Zea mays* (*Zm*), *Arabidopsis thaliana* (*At*), *Populus trichocarpa* (*Ps*), and *Vitis vinifera* (*Vv*) using hmmsearch (*E* < 1 × e^−5^) implemented in HMMER version 2.3.2 (http://hmmer.janelia.org/). The collected sequences were aligned using ClustalW v2.0 (http://www.ebi.ac.uk/Tools/webservices/services/msa/clustalw2_soap). PhyML v3.0 (http://www.atgc-montpellier.fr/phyml/) [48] was then used to construct phylogenetic trees by the maximum likelihood method under the Jones-Taylor-Thornton model [49] with default parameters, and the reliability of interior branches was assessed with 1000 bootstrap resamplings. Phylogenetic trees were displayed using MEGA v4.0 (http://www.megasoftware.net/mega4/mega.html) [50].

### 4.3. Isolation of Total RNA and Semi-Quantitative RT-PCR Analysis

Total RNA was extracted using Trizol reagent (Invitrogen, Carlsbad, CA, USA) according to the manufacturer’s protocol. The extracted RNA was treated with RNase-free DNaseI (TaKaRa, Dalian, China) to eliminate genomic DNA contamination according to the protocols recommended by the manufacturer. The first strand of cDNA was synthesized from 2.0 μg of total RNA using the M-MLV First Strand Kit (Invitrogen) and the cDNA products equivalent to 200 ng of total RNA were used as templates in a 25 μL PCR reaction system. Semi-quantitative RT-PCR analyses for gene expression were performed on a PCR instrument (S1000™ Thermal Cycler, BIO-RAD, Foster City, CA, USA). PCR primers used in semi-quantitative RT-PCR were designed using Primer Premier 6.0 software (http://www.premierbiosoft.com/primerdesign/index.html) to create PCR products spanning one to five exon(s) and the primer sequences are listed in Supplemental Table 1. The rice *Actin1* gene was used as an internal control in semi-quantitative RT-PCR analysis.

### 4.4. Real-Time qPCR Analysis

Real-time qPCR was performed with Platinum SYBR Green qPCR SuperMix-UDG with ROX (Invitrogen) on CFX96™ Real-Time PCR Detection System (BIO-RAD, Foster City, CA, USA). PCR was carried out with the two-step protocol as follows: preheating at 95 °C for 3 min, followed by 40 cycles of denaturation at 95 °C for 5 s and annealing/extension at 62 °C for 30 s. The expression levels of each gene were obtained by normalization to that of *OsActin1* and relative expressions were compared with that of control plants. Means values were obtained from three independent PCR amplifications. The primer sequences are listed in Table S2.

## 5. Conclusions

In summary, the expression profiles of rice *Nox* genes varied greatly with tissues and environmental changes, such as drought, heat, salt, and calcium, implying diverse functions of Noxs in the plant development and stress responses. The diversity of function is supported by the number of *Nox* genes, the observed differences in functional protein domains, as well as the unique patterns of gene expression changes in response to these four stressors and different organs. Different changes in expression profiles of the same *Nox* gene and different *Nox* genes to different environmental factors imply their close but not identical functions and/or regulatory mechanisms. The results presented here provide the groundwork for further experiments aimed at determining the exact role of each rice *Nox* gene in regulating stress responses as well as normal development, and for examining the potential for cross-talk between rice Nox proteins.

## Supplementary Information



## Figures and Tables

**Table 1:** NADPH oxidases (Noxs) and their predicted characters in rice *.

Gene name	Other names	Accession numbers	Gene locus		Protein size (predicted, aa)	Molecular weight (predicted, kD)	Sources

			Os ID	MSU’s LOC_Os ID			
*OsNox1*	*OsRbohB*	AY603975	Os01g0360200	LOC_Os01g25820	905	101.759	http://www.uniprot.org/uniprot/Q5ZAJ0
*OsNox2*	*OsRbohA*	NP_001044165.1	Os01g0734200	LOC_Os01g53294	745	85.336	http://www.uniprot.org/uniprot/O48539
*OsNox3*	*OsRbohE*	AK100241	Os01g0835500	LOC_Os01g61880	843	94.79	http://www.uniprot.org/uniprot/Q8S1T0
*OsNox4*	*OsRbohD*	AK072353	Os05g0465800	LOC_Os05g38980	819	92.35	http://www.uniprot.org/uniprot/Q0DHH6
*OsNox5*	*OsRbohC*	AK120905	Os05g0528000	LOC_Os05g45210	951	107.171	http://www.uniprot.org/uniprot/Q65XC8
*OsNox6*	*RbohE*	NP_001061956.1	Os08g0453700	LOC_Os08g35210	1033	115.014	http://www.uniprot.org/uniprot/Q0J595
*OsNox7*	*OsRbohG/OsRbohB*	NP_001063267.1	Os09g0438000	LOC_Os09g26660	1007	112.134	http://www.uniprot.org/uniprot/Q69LJ7
*OsNox8*	*OsRbohI*	AK063113	Os11g0537400	LOC_Os11g33120	936	72.025	http://rapdblegacy.dna.affrc.go.jp/viewer/gbrowse_details/build5?name=Os11g0537400
*OsNox9*	*OsRbohH*	J075145A22	Os12g0541300	LOC_Os12g35610	892	99.893	http://rice.plantbiology.msu.edu/cgi-bin/ORF_infopage.cgi?orf=LOC_Os12g35610.1
*OsFRO1*		AB126085	Os04g0578600	LOC_Os04g48930	537	58.095	http://www.uniprot.org/uniprot/Q0JAT2
*OsFRO7*		AK067009	Os04g0444800	LOC_Os04g36720	756	83.156	http://www.uniprot.org/uniprot/Q0JCX7

* Gene locus of *Noxs* from MSU rice genome annotation (http://rice.plantbiology.msu.edu/) and protein codes in NCBI (http://www.ncbi.nlm.nih.gov/) are presented. Two proteins, OsFRO1 and OsFRO7, which the most known functions are to act as ferric reduction oxidases, are also listed here since these two proteins were considered as ancient forms of Noxs and their encoding genes were grouped to rice *Nox* gene family in NCBI database.
