# Supplementary material for: Characterization of Rice NADPH Oxidase Genes and Their Expression under Various Environmental Conditions
Source: Int J Mol Sci. 2013 Apr 29;14(5):9440–58. doi: 10.3390/ijms14059440 (PMC3676792; doi:10.3390/ijms14059440)
Supplement: Supplementary file 1 [file ijms-14-09440-s001.pdf]

Supplementary Information

Table S1. The Primers used for semi-quantitative RT-PCR analysis.

Gene	Forward Primer (5'→3')	Reverse Primer (5'→3')	Product size	Cycle numbers	Exon numbers
OsNox1	AAGGGAATAACGGACGAAA	CCTCTGAACCACTCAAACG	296 bp	29	1
OsNox2	CACAACTACCTAACAAGCGTC	TCCTCACCTTGCTATCTCC	411 bp	34	3
OsNox3	CTTTTGCTTATTGGTCTTGG	TTGTTTCGTGAGTGTAGGG	477 bp	35	5
OsNox4	CAAGCGAGGTGTTTGTGGCA	GGCTGTCACCATACCACGGA	283 bp	34	4
OsNox5	TTACTGCTGGTTGGATTAGGA	CATAGTAATGAGTGCTGACCGA	366 bp	35	3
OsNox6	TCACCAAGTGGCACCAGAGG	GAACGCTTGGCACGAAATAG	360 bp	32	3
OsNox7	GTCAAATGCTTATGCTGTCA	TGTCCAGTCTCCGTTTGTT	223 bp	38	3
OsNox8	CCCAGCAACCTCGGCTACAT	ACGCAGACGCAGTAGCCCAT	314 bp	38	1
OsNox9	CCGTAAGGATTGAGAAGGT	GGGTCGTCGTAGATGTGGT	340 bp	45	3
OsFRO1	ATTGTGACAGGCATAGAACTCG	GACTGGAAAGAACAGGAACG	333 bp	38	1
OsFRO7	GCCTGCTAATGTGGGTGAC	GGAGACTTGCTGGCTTTGA	273 bp	36	1
OsActin1	CAGCACATTCCAGCAGATGT	TAGGCCGGTTGAAAACTTTG	198 bp	24	2

Table S2. Primers used for real-time qRT-PCR analysis.

Gene	Forward Primer (5'→3')	Reverse Primer (5'→3')	Exon numbers
OsNox1	GGCTTCAATGCCTTCTGGT	ATGGCTCCTAAACAACCGA	2
OsNox2	ATCCGCAAAATAAGCACCTCT	CAGTAGCCCATCACATCAAAGAC	1
OsNox3	TCAAGGCAGCGATTTACCC	CTCGCAAGCCTTCCCAAA	2
OsNox4	CACAAGGTTATCGCACTGACG	AGCGATGAGTATGTTGGTTGA	1
OsNox5	CCAGTGGGTGGGAAAAGTG	GTCCGATTGGCGGGTAAA	3
OsNox6	CCTTTCTCCATCACTTCAGCA	GGGCCATCTACAAGCAACC	2
OsNox7	GTCAAATGCTTATGCTGTCA	TGTCCAGTCTCCGTTTGTT	3
OsNox8	ACCTTACCTGCGATTTTCCA	ACGAAGCAGTGGTGGGAGT	1
OsNox9	TACTTCGGGCAGACACGGAT	GCGGGTTGCTGTCACTAAG3	1
OsFRO1	TCCTGTTCTTTCCAGTCGC	GTTCTTGTCCATTTCAGCATCT	1
OsFRO7	CTGGCATTTCACGTTGGC	CGGAGACTTGCTGGCTTTG	1
OsActin1	GTGGTCGCCCCTCCTGAAAG	GGCTTAGCATTCTTGGGTCCG	2

© 2013 by the authors; licensee MDPI, Basel, Switzerland. This article is an open access article distributed under the terms and conditions of the Creative Commons Attribution license (http://creativecommons.org/licenses/by/3.0/).
